# Cold atmospheric plasma induces GSDME-dependent pyroptotic signaling pathway via ROS generation in tumor cells

**DOI:** 10.1038/s41419-020-2459-3

**Published:** 2020-04-27

**Authors:** Xiaorui Yang, Guodong Chen, Kwan Ngok Yu, Miaomiao Yang, Shengjie Peng, Jie Ma, Feng Qin, Wei Cao, Shujun Cui, Lili Nie, Wei Han

**Affiliations:** 1 Anhui Province Key Laboratory of Medical Physics and Technology/Center of Medical Physics and Technology, Hefei Institutes of Physical Sciences, Chinese Academy of Sciences, Hefei, Anhui China; 2 University of Science and Technology of China, Hefei, Anhui China; 3 Department of Physics, City University of Hong Kong, Tat Chee Avenue, Kowloon Tong, Hong Kong; 4 State Key Laboratory in Marine Pollution, City University of Hong Kong, Tat Chee Avenue, Kowloon Tong, Hong Kong; 5 Clinical pathology center, The Fourth Affiliated Hospital of Anhui Medical University, Hefei, Anhui China; 6 Collaborative Innovation Center of Radiation Medicine of Jiangsu Higher Education Institutions and School for Radiological and Interdisciplinary Sciences (RAD-X), Soochow University, Suzhou, Jiangsu China

**Keywords:** Cancer therapy, Cell death

## Abstract

Cold atmospheric plasma (CAP) has been proposed as a novel promising anti-cancer treatment modality. Apoptosis and necrosis have been revealed in CAP-induced cell death, but whether CAP induces pyroptosis, another kind of programmed cell death is still unknown. In the present study, we first reported that CAP effectively induced pyroptosis in a dose-dependent manner in Gasdermin E (GSDME) high-expressed tumor cell lines. Interestingly, the basal level of GSDME protein was positively correlated with the sensitivity to CAP in three selected cancer cell lines, implying GSDME might be a potential biomarker of prognosis in the forthcoming cancer CAP treatment. Moreover, our study revealed that CAP-induced pyroptosis depended on the activation of mitochondrial pathways (JNK/cytochrome c/caspase-9/caspase-3) and the cleavage of GSDME but not Gasdermin D (GSDMD). ROS generation induced by CAP was identified to initiate the pyroptotic signaling. These results complemented our knowledge on CAP-induced cell death and provide a strategy to optimize the effect of CAP cancer treatment.

## Introduction

In fighting cancers, scientists constantly explore new physical technologies beyond ionizing radiation to kill cancer cells effectively. Cold atmospheric plasma (CAP), an ionized gas with a near room temperature, consists of reactive species, ions, electrons, neutral particles, ultraviolet, visible light, etc.^1^. Over the past decade, CAP treatments have been identified as a powerful and practical technique in anti-cancer therapy with the notable advantages that CAP could effectively and selectively kill various types of tumors cells and distinctly less damage to normal cells in vitro and in vivo^2–4^. Reactive oxygen species (ROS) have been considered as the major effectors in CAP-induced killing of tumor cells^5,6^. Numerous studies have revealed that CAP exposure increase the level of intracellular ROS, and cause DNA damage, cell cycle arrest, mitochondria damage etc., then finally induce apoptosis or necrosis of tumor cells^7–10^. As far as we know, ROS can trigger apoptosis, necrosis, ferroptosis, pyroptosis and other types of cell death^11–15^. However, it remains unclear whether CAP treatment can induce other types of cell death in addition to apoptosis and necrosis.

Pyroptosis, a type of lytic programmed cell death (PCD), is characterized by cell swelling with large bubbles bulging from the plasma cytoplasmic membrane and cell lysis, leading to the release of pro-inflammatory molecules^16^. The early studies identified a pyroptosis executioner, gasdermin D (GSDMD), which was cleaved after the activation of caspase-1 and caspase-11/4/5 in immune cells^17,18^. The pyroptotic N-terminal fragment of GSDMD binds lipids and forms membrane pores, which trigger cell swelling and membrane rupture^19,20^. Recently, another gasdermin family member, gasdermin E (GSDME), was reported to induce pyroptosis in various cancer cells^15,21–24^. Different from GSDMD, GSDME is cleaved by activated caspase-3 to generate a GSDME-N fragment, which executes pyroptosis by forming pores in the plasma membrane^25,26^. In addition, GSDME also has been identified as a possible tumor suppressor gene^27,28^, and epigenetic silencing through GSEME methylation has been found in gastric, colorectal, and breast cancer samples^28–30^. Furthermore, loss of GSDME confers the resistance to etoposide in melanoma cells^31^. In short, these studies imply that GSDME is a new potential contributor to cancer cell death.

Herein, we firstly revealed that CAP, as one physical factor but not the conventional chemical or biological ones, induced GSDME-mediated pryoptosis in tumor cells and the basal level of GSDME was positively correlated to the sensitivity to CAP treatment. Additionally, our results also showed that ROS/Caspase-9/caspase-3 apoptotic pathway was activated by CAP exposure to cause the cleavage of GSDME and then led to pyroptosis. Our study provided new insights into the mechanism for cancer cell death induced by CAP and proposed a new strategy to evaluate the sensitivity and to optimize the effect of CAP treatment.

## Materials and methods

### Cell culture, reagents, and antibody

A549, PC9 (human lung carcinoma), SGC7901 (human gastric carcinoma), and Bel7402 (human hepatoma carcinoma) cells were purchased from the American Type Culture Collection (ATCC). Other cells were obtained from the Type Culture Collection of the Chinese Academy of Sciences. H1299, MKN28 and SGC7901 cells were cultured in RPMI 1640 medium (RPMI 1640, Hyclone, Logan, USA) supplemented with 10% Fetal Bovine Serum (FBS, Thermo Scientific Hyclone, Logan, UT, USA) and 1% penicillin/streptomycin (Gibco, Carlsbad, CA, USA). Other cells were cultured in high glucose Dulbecco’s modified Eagle medium (DMEM, Hyclone, Logan, USA) supplemented with 10% FBS and 1% penicillin/streptomycin. All cells were maintained in a humidified incubator under 5% CO_2_ at 37 °C and routinely checked for mycoplasma contamination. All cell lines used in this study were authenticated by short tandem repeats (STRs) profiling.

Caspase inhibitor (Z-VAD-FMK) and casaspe-9 inhibitor (Z-LEHD-FMK) were purchased from Selleck Chemicals (Houston, TX, USA). 3-(4,5-dimethylthiazol-2-yl)-2,5-diphenyl tetrazolium bromide (MTT) and N-acetyl-L-cysteine (NAC) were purchased from Beyotime Biotechnology (Shanghai, China). Puromycin was purchased from Gibco (Life Technologies, Carlsbad, CA, USA). The primary antibodies against GSDMD (Cat# 96458S), Caspase-9 (Cat# 9502S), Caspase-8 (Cat# 9746S), Caspase-3 (Cat# 14220S), PARP (Cat# 9532S), Bax (Cat# 2772S), JNK (Cat# 9258S) and phosphorylated JNK (p-JNK, T183/Y185) (Cat# 4668S) were purchased from Cell Signaling Technology (Danvers, MA, USA). The primary antibodies against cleaved GSDME (Cat# ab215191) and cytochrome c (Cat# ab50050) were purchased from Abcam (Cambridge, MA, USA) and β-actin (Cat# 66009-1-lg), β-Tublin (Cat# 66240-1-lg) and COX IV (Cat# 11242-1-lg) primary antibodies were purchased from Proteintech (Wuhan, China). Secondary IRDye-labeled goat anti-mouse and anti-rabbit IgG antibodies were purchased from LI-COR Biosciences (LI-COR, Lincoln, NE, USA).

### CAP treatment

The CAP generator consists of a hollow plexiglass as a reactor chamber with four electrodes, one air inlet and one outlet, as described in our previous studies^32,33^. The high voltage electrode was a 32 mm diameter copper cylinder, covered by 1 mm thick quartz glass as an insulating dielectric barrier. The ground electrode was a 37 mm diameter copper cylinder. CAP was generated by a voltage of 12 kV (peak to peak) with a frequency of 24 kHz. The discharge power density was measured to be about 0.9 W/cm^2^. The discharge gap between the bottom of the quartz glass and medium surface was maintained at 5 mm. Helium gas (99.99% pure) was used as the working gas with a flow rate 120 L/h, and injected 3 min before discharging to expel air as much as possible from reactor chamber. Cells were seeded in Petri dishes (35 mm diameter) with 2 mL complete culture medium overnight and three Petri dishes from each group were randomly selected. For CAP exposures, cells were exposed to CAP for predicted time, which determined the dose of CAP.

### Cell viability assay

At 24 h after CAP exposure, the cells were treated with MTT solution (0.5 mg/mL, Biofroxx, Einhausen, Hessen, Germany) for 4 h at 37 °C, and then 100 μL of MTT formazan solution in DMSO was transferred into the 96-well plates. The optical density (OD) values were measured at 490 nm by using a Varioskan Flash microplate reader (Thermo Fisher Scientific, Rockford, IL, USA).

### LDH release assay

The activity of lactate dehydrogenase (LDH) released into cell culture supernatants was measured with CytoTox 96 Non-Radioactive Cytotoxicity Assay Kit (Promega, Madison, WI, USA) according to the manufacturer’s protocol. The absorbance value at 450 nm was then measured.

### Cell death detection with flow cytometery

CAP-induced cell death was detected with Annexin V-FITC/PI or Annexin-APC/PI apoptosis detection kit (BD Biosciences, Bedford, MA, USA) according to the manufacturer’s protocol. Briefly, the cells were harvested at 24 h after CAP treatment and incubated with Annexin V-FITC/PI or Annexin-APC/PI for 15 min at room temperature in dark. The cells were then immediately analyzed with a flow cytometer (Accuri C6, BD Biosciences, Bedford, MA, USA). All the data analyses were performed with FlowJo analysis software (TreeStar, Ashland, OR, USA).

### Western blot

Cells were lysed in RIPA buffer (Beyotime Biotechnology, Shanghai, China), and the protein concentration was determined with BCA Protein Assay Reagent Kit (Beyotime Biotechnology, Shanghai, China). The mitochondrial and cytoplasmic proteins were separated with the cytoplasmic and mitochondrial protein extraction kit (Sangon Biotech, Shanghai, China). Equal amounts of protein extracts (45 μg) were subjected to SDS-PAGE, then transferred onto polyvinyl difluoride (PVDF) membranes (Millipore Corporation, Bedford, MA, USA) and blocked with 5% non-fat dry milk for 1 h at room temperature. The membranes were incubated with primary antibodies at 4°C overnight. After washing three times with TBST (0.1% Tween-20 in Tris-HCl buffer), the membranes were incubated with IRDye-labeled secondary antibodies for 1 h at room temperature. Protein bands were visualized with an Odyssey® CLx Infrared Imaging System (LI-COR, Lincoln, NE, USA).

### ROS measurement

The intracellular ROS were measured with fluorescent probe DCFH-DA (Beyotime Biotechnology, Shanghai, China) or dihydro-ethidium (DHE) (Invitrogen, Carlsbad, CA, USA) following the manufacturer’s instruction. At 12 h after CAP exposure, the cells were stained with DCFH-DA solution (10 µM) for 30 min or DHE solution (5 µM) for 1 h at 37 °C in dark and then washed three times with PBS. The fluorescence was determined with a fluorescence microscope (Leica DMI 4000B, Germany) or a flow cytometer (Accuri C6, BD Biosciences, Bedford, MA, USA).

### Generation of stable GSDME knockdown and overexpression cell lines

For silencing of GSDME, the shRNA target sequences were used as following: TGATGGAGTATCTGATCTT (RNAi 1#) and ATTCATAGACATGCCAGAT (RNAi 2#), and both shRNAs were subcloned into GV248 lentiviral vectors (GenePharma, Shanghai, China). The lentiviruses were generated by transfecting HEK293T cells together with the lentiviral vector and packaging plasmids. At 48 h after transfection, the viral supernatants were collected to infect PC9 cells. Cells were selected with puromycin (0.75 μg/mL) for 5 days and then tested for GSDME expression by western blotting analysis.

For GSDME stable overexpression, human GSDME overexpression plasmids (a kind gift from Dr. F. Shao, National Institute of Biological Sciences, Beijing, China), constructed by inserting cDNAs of human gasdermin E into a modified pWPI lentiviral vector with an N-terminal 2×Flag-HA^26^, were packaged into lentivirus particles. Virus-containing supernatant was collected at 48 h after transfection and then infected H1299 cells in 6-well dishes. At 72 h after viral infection, the cells were sorted for GFP expression with flow cytometry (BD FACSCalibur, BD Biosciences, USA), and then the expression of GSDME was detected with western blotting.

### siRNA Transfection

Specific siRNAs for Caspase-9 (sense: 5′CAGUAUCGCUCAUAGAUCATT 3′ and antisense: 5′UGAUCUAUGAGCGAUACUGTT3′), Caspase-3 (sense: 5′UGAGGUAGCUUCAUAGUGGTT 3′ and antisense: 5′CCACUAUGAAGCUACCUCATT 3′) and the control siRNA (sense: 5′ UUCUCCGAACGUGUCACGUTT 3′ and antisense: 5′ ACGUGACACGUUCGGAGAATT 3′) were synthesized by GenePharma Co. Ltd (Shanghai, China). The cells were transfected with double-stranded siRNAs with the Lipofectamine®2000 transfection reagent (Invitrogen, Carlsbad, CA, USA) according to the manufacturer’s protocols. At 48 h after transfection, the cells were exposed to CAP and the proteins were collected at the indicated time points for further experiments.

### Statistical analysis

Statistical analysis was performed on the data obtained from at least three independent experiments, each with three replicates. Statistical analysis was performed using Graph Pad Prism 6 (GraphPad Software, Inc., LaJolla, CA, USA). The data were represented as the mean ± SD and analysis of variance or two-tailed Student’s *t* test were used for statistical comparison to determine significance. *P* < 0.05 was considered a statistically significant difference (NA, *p* > 0.05; *p < 0.05; **p < 0.01; ***p < 0.001).

## Results

### Basal level of GSDME tightly associated with CAP sensitivity

**Fig. 1:**
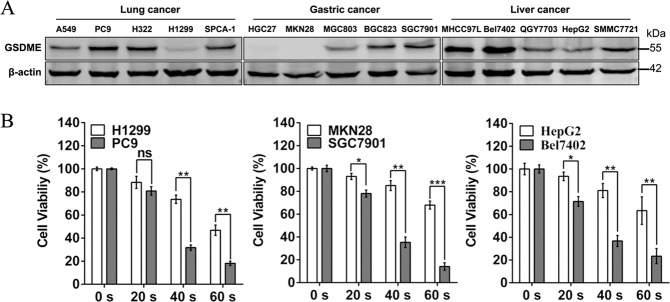
The CAP sensitivity is associated with the expression level of GSDME in three types of cancer. **a** The expression of endogenous GSDME in 15 tumor cell lines derived from three types of cancer was determined by western blotting. **b** The cell viability was detected at 24 h after CAP exposures for 20–60 s in the indicated cells with high/low GSDME expression. *p < 0.05, **p < 0.01, ***p < 0.001.

The basal expression of GSDME in 15 human tumor cell lines, derived from lung cancer (A549, PC9, H322, H1299, and SPCA-1), gastric cancer (HGC27, MKN28, MGC803, BGC823 and SGC7901) and liver cancer (MHCC97L, Bel7402, QGY7703, HepG2, and SMMC7721), were determined. Results in Fig. 1a showed that the basal levels of GSDME significantly varied in the selected tumor cell lines. GSDME was present in all 10 lung and liver cancer cell lines, but absent in two (HGC27 and MKN28) of five gastric cancer cell lines. To assess the relationship between expression of GSDME and CAP sensitivity, three pairs of cell lines (PC9/H1299, SC7901/MKN28 and Bel702/HepG2) with relatively high or low GSDME expression were selected and tested for the cell viability with MTT at 24 h after CAP exposure. The results showed that the viability of PC9, with high GSDME expression level, was significantly lower than that of H1299 with low GSDME level after same dose CAP exposure (Fig. 1b). Similar results were also observed in the pairs of gastric and liver cell lines (Fig. 1b). These results indicated that the level of basal GSDME protein had positive correlation with the cell sensitivity to CAP exposure.

### CAP induce pyroptosis in GSDME high-expressed tumor cells in a time- and dose-dependent manner

**Fig. 2:**
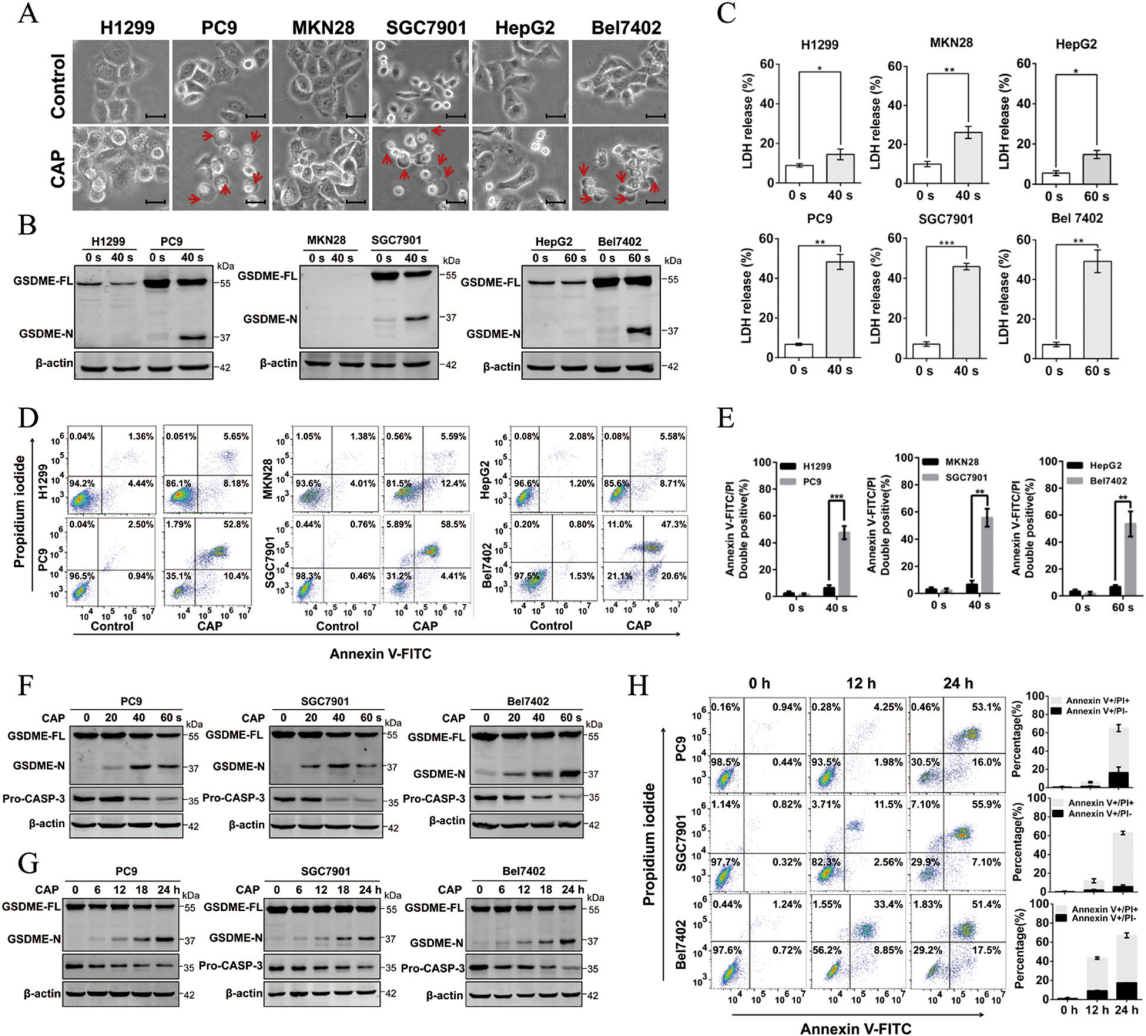
CAP can induce cell pyroptosis in lung, gastric and liver cancer cells. **a**–**c** The features of cell pyroptosis were detected at 24 h after CAP treatment in three pairs of high/low GSDME expression cell lines as indicated. **a** Representative bright-field microscopy images in which red arrowheads indicated the large bubbles emerging from the plasma membrane. Scale bar, 25 µm. **b** Full-length GSDME (GSDME-FL) and GSDMD-N terminal (GSDMD-N) detected by western blotting. **c** Release of LDH in culture supernatants. **d** Annexin V-FITC/PI assay was performed to identify pyroptosis and apoptosis cells after CAP treatment in three pairs of high/low GSDME expression cell lines as indicated. **e** Quantification of pyroptotic cells double stained with annexin V-FITC/PI. **f** Western blot analyses of expression of GSDME-FL, GSDME-N and Pro-CASP-3 (pro-caspase-3) were performed at 24 h after indicated CAP exposure dose in GSDME high-expressed cell lines. **g** Western blot analyses of expression of GSDME-FL, GSDME-N and Pro-CASP-3 were performed at indicated incubation time after CAP exposure in GSDME high-expressed cell lines. **h** Cell death was assessed by measuring annexin V-FITC- and PI-stained cells at indicated time after CAP exposures in GSDME high-expressed cell lines. (Left: representative flow cytometric dotplots; right: quantification of pyroptotic cells double stained with annexin V-FITC/PI). All data are presented as the mean ± SD from three independent experiments. *p < 0.05, **p < 0.01, ***p < 0.001.

For further comparison purposes, the selected cell lines (PC9/H1299, SC7901/MKN28 and Bel702/HepG2) were treated with CAP (40 or 60 s) and incubated for 24 h. After CAP treatment, large bubbles from the plasma membrane and cell swelling, morphological features of pyroptosis, were observed frequently in GSDME high-expressed PC9, SGC7901 and Bel7402 cells, but rarely in GSDME low-expressed H1299, MKN28, and HepG2 cells (Fig. 2a). Moreover, the cleavage of GSDME, another characteristic pyroptotic marker, was induced in CAP-treated cells with high GSDME expression (PC9, SGC7901, and Bel7402) (Fig. 2b), suggesting that it was likely that CAP specifically induced cell death through pyroptosis in GSDME high-expressed cells. These results were confirmed by detecting the release of LDH and the percentage of annexin V and PI double-positive cells for cellular membrane integrity loss and leakage during pyroptosis. More LDH release was detected in GSDME high-expressed tumor cells than low-expressed cells after exposing to the same CAP dose (Fig. 2c). In addition, in GSDME high-expressed tumor cells, a majority of dead cells after CAP treatment exhibited pyroptotic characteristics, showing double positive for annexin-V and PI, and only a small portion of cells underwent apoptosis (annexin-V-positive but PI-negative) (Fig. 2d, e). However, GSDME low-expressed cells subjected to 40 or 60 s exposure showed opposite trends, i.e., a relatively high proportion of apoptotic cells (annexin-V-positive but PI-negative) and a relatively low proportion of cells with double positive for annexin-V and PI (Fig. 2d, e and Supplementary Fig. 1). Taken together, these results further supported the findings that CAP could induce pyroptosis associated with GSDME.

Furthermore, we explored the dose and time dependence of CAP-induced pyroptosis in GSDME high-expressed PC9, SGC7901 and Bel7402 cells. We found that the cleavage of GSDME increased gradually along with elevated CAP dose (Fig. 2f, g). Additionally, the LDH release and the percentage of annexin V/PI double positive cells also increased with the prolonged incubation time after CAP exposure (Fig. 2h–j). These data together revealed that CAP induced pyroptosis in a time- and dose-dependent manner.

Combining all of aforementioned results, we identified that CAP triggered pyroptosis in GSDME high-expressed tumor cells and the pyroptosis showed dose and time dependence.

### GSDME-mediated CAP-induced pyroptosis in tumor cells

**Fig. 3:**
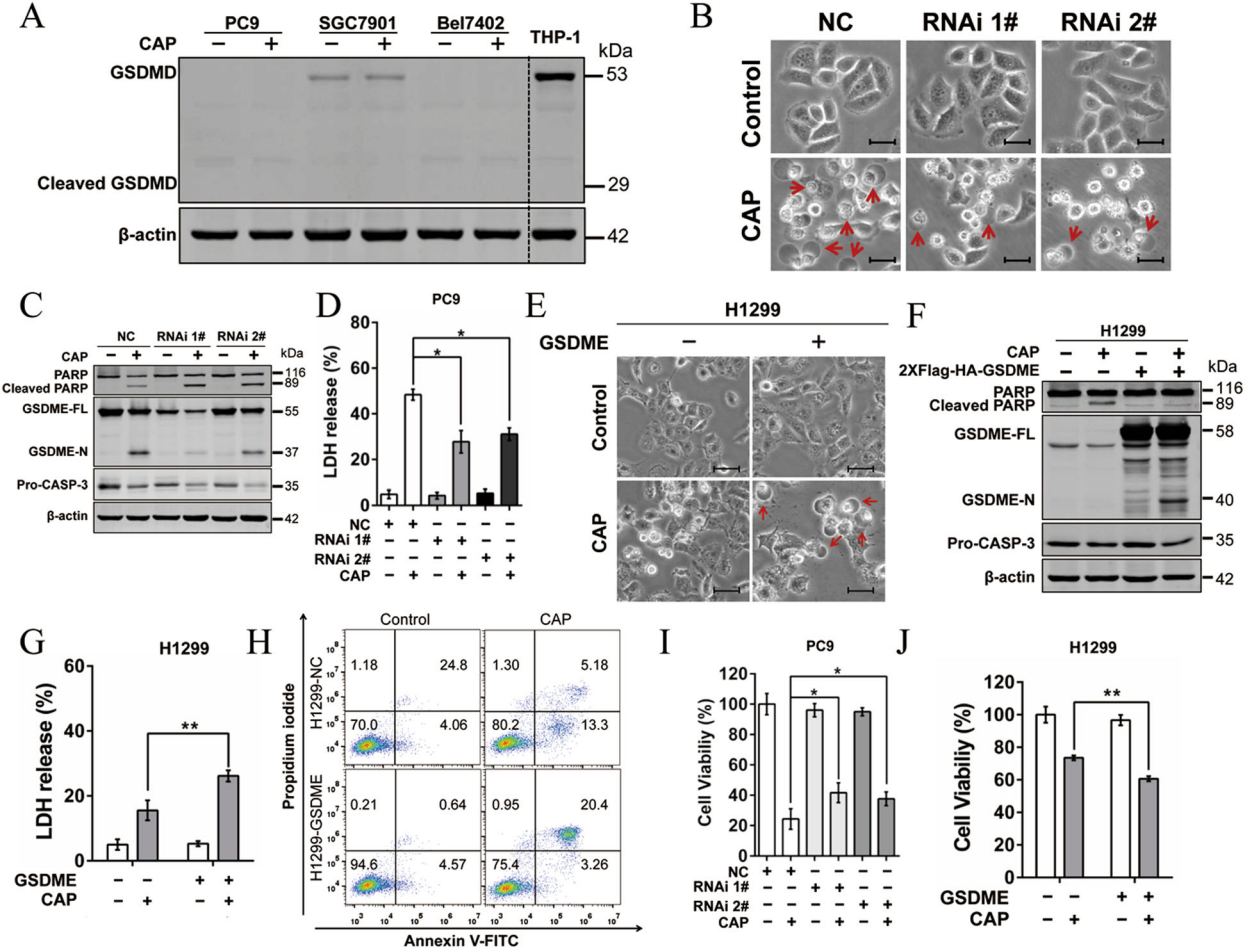
GSDME is essential in CAP-induced pyroptosis. **a** GSDMD-FL and cleaved GSDMD (GSDMD-N) were detected by western blotting in PC9, SGC-7901 and Bel7402 at 24 h after CAP exposures. THP1 cells expressing GSDMD were used as a positive control. **b**–**d** Alterations in features of cell pyroptosis were determined upon CAP treatment after knocking down GSDME in PC9 cells. **b** Representative microscopic images of negative control (NC) and GSDME-knockdown PC9 cells (RNAi#1 and RNAi#2). Red arrowheads indicated large bubbles emerging from the plasma membrane. Scale bar, 25 µm. **c** The apoptosis- and pyroptosis-related proteins including PARP, cleaved-PARP, GSDME, GSDME-N and pro-CASP-3 analyzed by western blotting. β-actin served as loading control. **d** LDH release in the culture supernatants. **e**–**g** The features of cell pyroptosis were determined upon CAP treatment after overexpressing GSDME in H1299 cells. **e** Representative microscopic images. Red arrowheads indicated large bubbles emerging from the plasma membrane. Scale bar, 25 µm. **f** Apoptosis- and pyroptosis-related proteins including PARP, cleaved-PARP, GSDME, GSDME-N and Pro-CASP-3 determined by western blotting. **g** LDH release assays. **h** Annexin V-FITC/PI assay was performed to identify the pyroptotic and apoptotic cells after CAP treatment in H1299 and H1299-GSDME cells. **i**, **j** Cell viability was measured at 24 h after CAP exposures in GSDME knockdown PC9 cells (**i**) and GSDME overexpressed H1299 cells (**j**), respectively. All the data are presented as the mean ± SD from three independent experiments. *p < 0.05.

Numerous reports have demonstrated that GSDMD or GSDME function as the pyroptotic executioner^17,34^. To test whether GSDMD involved in CAP induced pyroptotic, full-length GSDMD and GSDMD-N (cleaved N-terminal of GSDMD) were detected after CAP treatment in PC9, SGC7901, and Bel7402. We found that the expression of GSDMD was nearly silent in PC9 and Bel7402 cells. Although GSDMD expressed at a low level in SGC7901 cells, no cleaved GSDMD was observed (Fig. 3a). Therefore, GSDME but not GSDMD was involved in CAP-induced pyroptosis. To further confirm the critical role of GSDME in CAP-induced pyroptosis, GSDME was stably knocked down in PC-9 cells. Unlike the pyroptotic morphology exhibited in the CAP-treated NC (negative control) cells, knockdown of GSDME resulted in lessened cell swelling (Fig. 3b), decreased GSDME-N protein level (Fig. 3c), and reduced LDH release (Fig. 3d) in response to CAP, indicating GSDME was necessary in CAP-induced pyroptosis. This idea was further supported by our findings that overexpression of GSDME promoted CAP-induced pyroptosis, displayed as more cell swelling (Fig. 3e), elevated GSDME-N protein level (Fig. 3f), more LDH release (Fig. 3g) and more annexin V/PI double-positive cells (Fig. 3h) in GSDME-overexpressing H1299 cells than H1299 cells upon CAP stimulation. In addition, overexpression of GSDME in H1299 cells switched CAP-induced apoptosis to pyroptosis (Fig. 3e, f, h), suggesting GSDME plays a key role in the switch of apoptosis to pyroptosis induced by CAP. Moreover, knockdown of GSDME in PC9 cells partly attenuated CAP-induced cell death (Fig. 3i), while overexpression of GSDME in H1299 cells promoted the CAP-induced cell death (Fig. 3i). These results suggested that pyroptosis, mediated by GSDME, might contribute to the cell sensitivity to CAP.

### Activation of Caspase-9/Caspase-3 was essential for CAP-induced pyroptosis

**Fig. 4:**
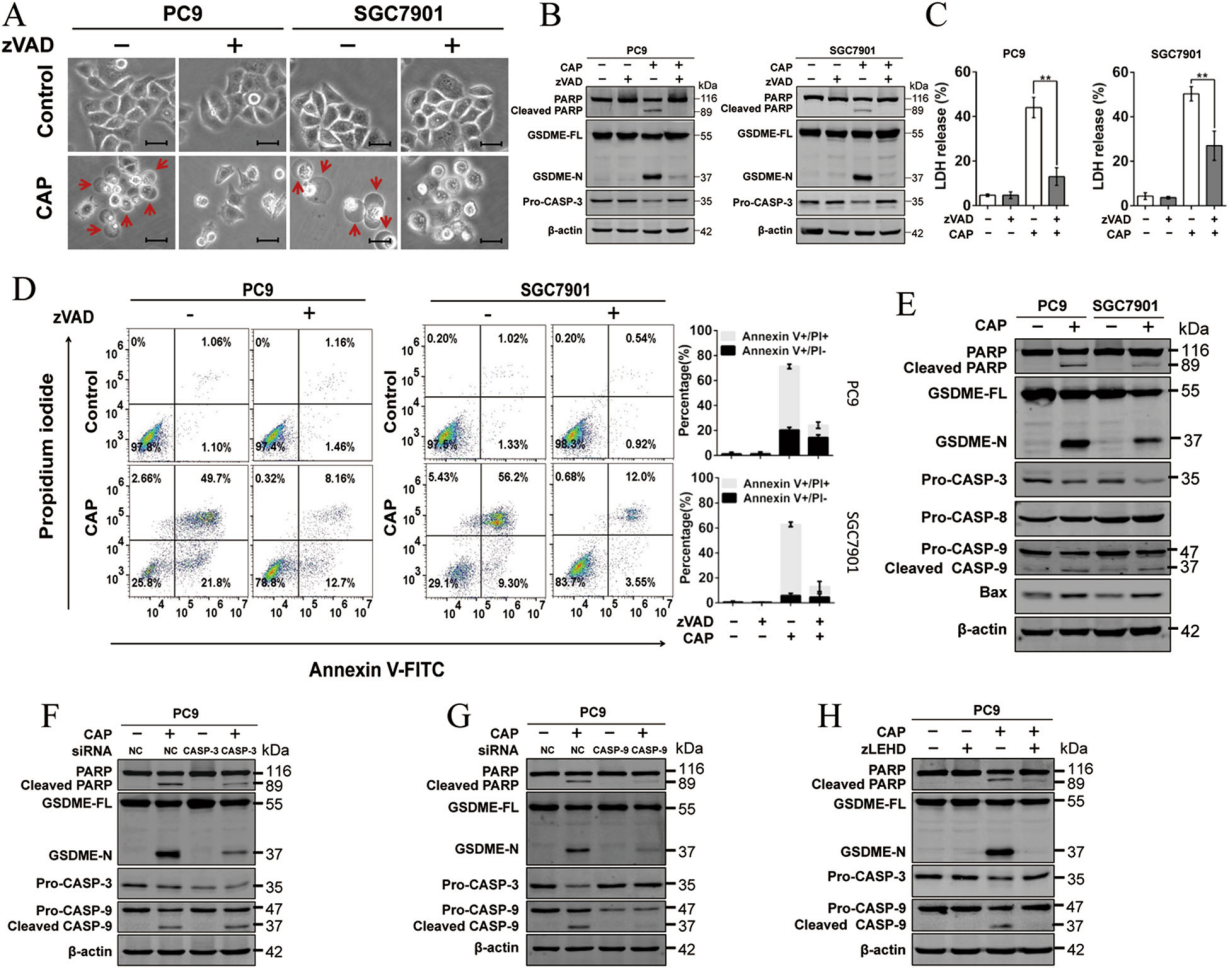
Activation of the caspase-9/caspase-3 pathway triggers the GSDME-mediated pyroptosis in response to CAP treatment. **a**–**d** The CAP-induced pyroptosis was repressed in PC9 and SGC-7901 cells pre-treated with pan-caspase inhibitor zVAD (30 µM) for 2 h following 40 s CAP exposures. **a** Representative microscopic images in which red arrowheads indicated large bubbles emerging from the plasma membrane. Scale bar, 25 µm. **b** Apoptosis- and pyroptosis-related proteins including PARP, cleaved-PARP, GSDME, GSDME-N and pro-CASP-3 detected by western blotting. **c** Release of LDH in the culture supernatant. **d** Cell death assessed by measuring annexin V-FITC- and PI-stained cells. **e** Apoptosis and pyroptosis-related proteins as indicated were detected after CAP treatment by western blotting in PC9 and SGC-7901cells. **f**, **g** Knocking down of caspase-3 (CASP-3) or caspase-9 (CASP-9) reduced the occurrence of apoptosis and pyroptosis induced by CAP exposure. Apoptosis and pyroptosis-related proteins as indicated were detected at 24 h after CAP exposures for 40 s in PC9 cells transfected with caspase-3 siRNA (**f**) and caspase-9 siRNA (**g**), respectively. **h** Apoptosis and pyroptosis-related proteins as indicated were detected at 24 h after CAP exposures for 40 s in PC9 cells pretreated with caspase-9-specific inhibitor zLEHD (30 µM). All the data are presented as the mean ± SD from three independent experiments. *p < 0.05, **p < 0.01.

To investigate the molecular mechanisms underlying GSDME-mediated pyroptosis induced by CAP exposure, we examined the activation of upstream caspases, which have been linked to the cleavage of GSDME^24,26^. PC9 cells were pre-treated with a caspase inhibitor, Z-VAD-FMK, and then exposed to CAP. An obvious attenuation was observed in terms of cell swelling (Fig. 4a), GSDME cleavage (Fig. 4b), LDH release (Fig. 4c) and cell death (Fig. 4d and Supplementary Fig. 2) comparing with Z-VAD-FMK untreated cells, confirming the involvement of caspase activation. Further dissection of upstream signaling molecules revealed that CAP effectively induced the activation of Bax, caspase-9 and -3 but not caspase-8 in GSDME high expressed-PC9 and GSC7901 (Fig. 4e). We next explored the exact role of caspases *via* blocking the function of caspase-9 and caspase-3, respectively. Knocking down either caspase-3 or -9 resulted in the reduction of GSDME-N (Fig. 4f, g) and caspase-9 knockdown inhibited the activation of caspase-3 (Fig. 4g), whereas loss of caspase-3 had no effect on caspase-9 activation (Fig. 4f). Consistent results were obtained by using caspase-9-specific inhibitor zLEHD-FMK (zLEHD) (Fig. 4h), confirming that caspase-9 was responsible for activation of caspase-3 in CAP-induced pyroptosis. Taken together, these results indicated that caspase-9/caspase-3/GSDME axis contributed to CAP-induced tumor cell pyroptosis.

### ROS initiated pyroptosis signaling after CAP exposure

**Fig. 5:**
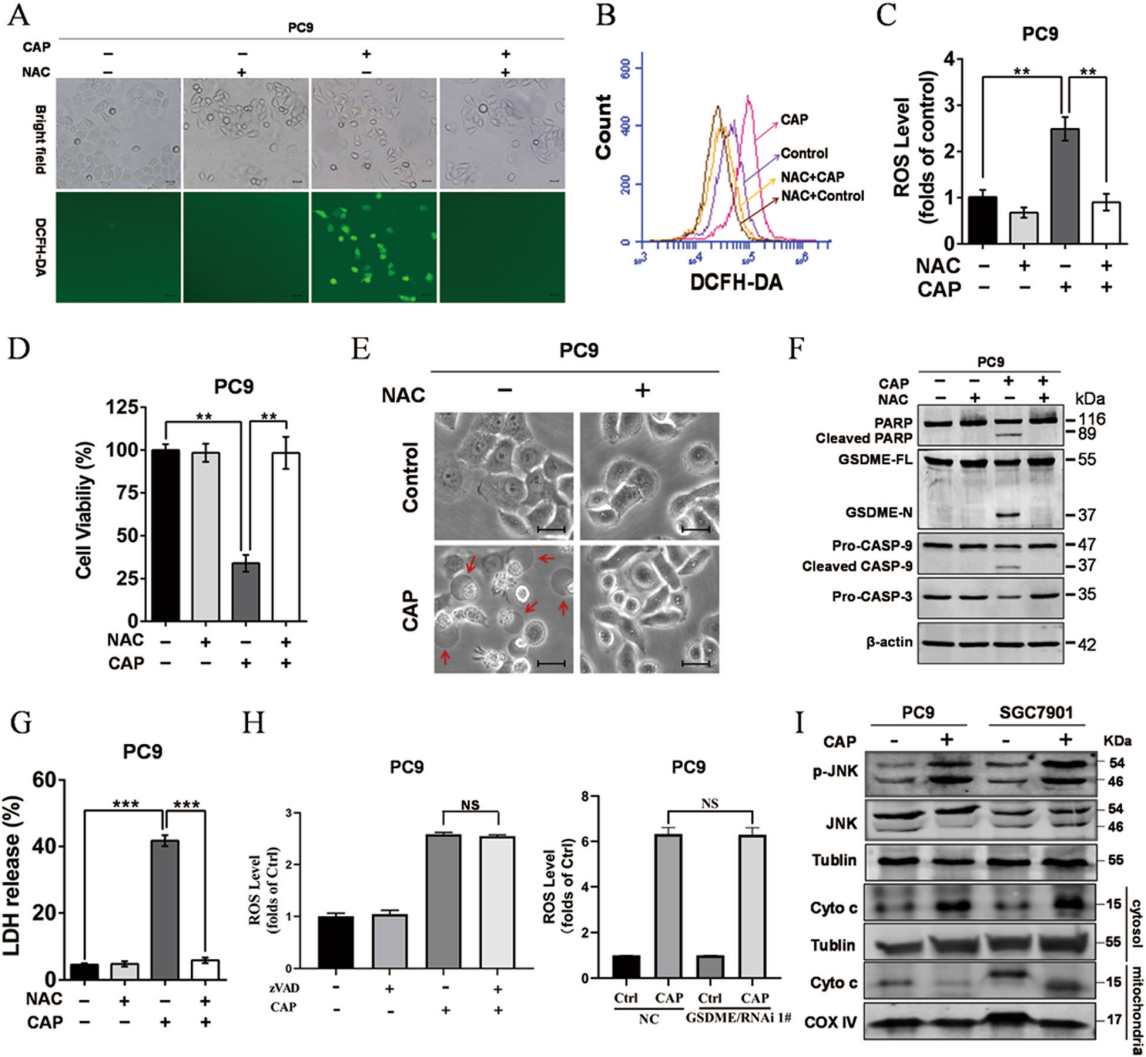
ROS initiates CAP-induced pyroptosis. **a**–**c** ROS in PC9 cells was detected after CAP exposure in the presence or absence of NAC (5 mM). **a** Representative bright-field and fluorescent images. **b** Representative flow cytometry histogram. **c** Qualitative flow cytometric analysis. **d** The cell viability was measured at 24 h after CAP treatment in the presence or absence of NAC. **e**–**g** The features of cell pyroptosis were determined at 24 h after CAP treatment in the presence or absence of NAC (5 mM). **e** Representative bright-field microscopic images in which red arrowheads indicated large bubbles emerging from the plasma membrane. Scale bar, 25 µm. **f** Apoptosis- and pyroptosis-related proteins as indicated detected by western blotting. **g** Release of LDH in culture supernatants. **h** CAP-induced ROS production was evaluated after zVAD treatment or knockdown of GSDME. **i** Phosphorylation of JNK and release of cytochrome c from mitochondria were detected by western blotting. All the data are presented as the mean ± SD from three independent experiments. *p < 0.05, **p < 0.01 and NS means no significant difference.

ROS have been reported to initiate apoptosis and necroptotic following CAP treatment^10^. However, whether CAP-induced ROS was linked to pyroptosis has not been reported. Indeed, our results also showed CAP treatment induced significant increase of ROS, which was reflected by the fluorescence of DCFH-DA (Fig. 5a, b). Of note, treatment with NAC, a scavenger of ROS, markedly reduced ROS production (Fig. 5a–c) and significantly elevated the cell viability after CAP exposure (Fig. 5d). Moreover, NAC treatment nearly completely attenuated the change of pyroptotic morphology (Fig. 5e), GSDME cleavage, caspase-3 activation (Fig. 5f) and LDH release (Fig. 5g), indicating removal of ROS effectively blocked CAP-induced pyroptosis. Meanwhile, CAP-induced ROS production was not affected by zVAD treatment or GSDME knockdown (Fig. 5h), suggesting that ROS acted as the upstream of the caspase-9/caspase3/GSDME signaling pathway. These results implied that ROS was the primary cause of tumor cell pyroptosis following CAP exposure. In addition, phosphorylation of JNK and increase of cytoplasmic cytochrome c were observed after CAP exposure (Fig. 5i), and these results suggested that JNK/ cytochrome c pathway played a key role in mediating the activation of ROS-dependent caspase-9. Taken together, our findings suggested CAP-induced ROS activated the JNK/cytochrome c/caspase-9/caspase3 pathway, and then cleaved GSDME to cause tumor cell pyroptosis subsequently.

## Discussion

CAP treatment has attracted attention as a potential strategy in cancer therapy for its multiple advantages^2–6^. Numerous studies have provided evidence that CAP treatment could effectively induce apoptosis in tumor cells^7,9–11^. As one kind of potential physical means in cancer therapy, whether CAP induce other types of cell death in tumor cells remains unclear. Recent studies have identified pyroptosis, another type of programmed cell death, may provide possible beneficial effect on anticancer therapies^17,26^. Furthermore, pyroptosis is also defined as gasdermin-mediated programmed necrosis since the gasdermin family members are indispensable executors of pyroptosis^16^. In our study, GSDME, one of gasdermin family members which are known as a prerequisite for pyroptosis occurrence, was found to be highly expressed in some tumor cells (Fig. 1A). These results were consistent with the previous study which showed overexpression of GSDME was present not only in normal cells but in some tumor cells including lung cancer, gastric carcinoma and melanoma^15,21,24^. Recently, both GSDMD and GSDME were reported to be critical effectors of pyroptosis occurrence^19,34^. In the present study, we observed GSDME-N but not GSDMD-N was generated together with other characteristics of pyroptosis including plasma membrane swelling and LDH release after CAP treatment in tumor cells (Figs. 2 and 3a). These results confirmed the previous report that GSDMD-dependent pyroptosis occurred mainly in immune cells^16,35^. Therefore, it could be inferred that CAP induced GSDME-mediated pyroptosis. This was further supported by our results that CAP-induced pyroptosis was decreased by knocking down GSDME, but increased by overexpression of GSDME (Fig. 3). Taken together, we revealed that CAP also distinctly induced typical GSDME-mediated pyroptosis in tumor cells. Although GSDME expressed in many normal tissues was associated with chemotherapy-induced tissue damage^26^, previous studies proved that CAP effectively and selectively killed various types of tumors cells and inflicted distinctly less damage to normal cells^2–4^. Moreover, CAP provided a kind of local treatment without systemic side effects and might be employed as a more promising tumor treatment via inducing GSDME-mediated pyroptosis. Hence, this study expanded our knowledge of CAP-induced cell death and offered new insights into CAP cancer therapy.

Excessive production of ROS led to several types of cell death including apoptosis^9^, necrosis^10,13^, autophagic cell death^12^, ferroptosis^14^ and pyroptosis^15,23^. Indeed, CAP treatment induced apoptotic or necroptotic *via* generating intracellular ROS^11^. In this study, our results showed that CAP treatment increased the production of ROS distinctly, and scavenging ROS with NAC effectively elevated the cell viability after CAP treatment, and even completely protected the cells against cell death at 5 μM with no increase of ROS (Fig. 5a–c, f). These studies were consistent with recent report that production of ROS induced by CAP initiated anticancer properties of CAP treatment^2,6^. Importantly, a further study showed that NAC treatment also blocked the cleavage of caspase-3 (Fig. 5e), which in turn could regulate the apoptosis or pyroptosis pathway^36^. Indeed, CAP-induced pyroptosis was inhibited after scavenging ROS with NAC (Fig. 5d, e, g), suggesting that ROS initiated pyroptosis signaling after CAP exposure. These studies were in agreement with a recent report that ROS signaling amplified by iron could induce the GSDME-mediated pyroptosis of melanoma cells^15^. In addition, ROS generation was also known to trigger GSDMD-mediated pyroptosis in macrophage^37^. Therefore, a sufficient amount of ROS may be an important initiator of pyroptosis in cells with high expression of GSDMD or GSDME.

Multiple types of death can be observed simultaneously in tissues or cell cultures after exposure to the same stimulus. In fact, our study also showed both apoptosis and pyroptosis were simultaneously observed after CAP treatment in PC9 cells, supported by the cleavage of both GSDME and PARP (Fig. 3c). The previous investigations revealed apoptosis and GSDME-mediated pyroptosis shared many signal transduction pathways, including involvement of caspase-3, caspase-8 and caspase-9^34,38^. Caspase-3 is known to be activated by caspase-9 (mitochondrial pathways) and caspase-8 (death receptor pathways), respectively^39^. Apoptosis can be initiated either through the death-receptor or the mitochondrial pathway. The former is initiated by various death stimuli or viral infection, which leads to permeabilization of the outer mitochondrial membrane causing cytochrome c release and further caspase-9 activation^40^. Death receptor pathway is activated by death receptor ligands at the cell membrane^41^. Indeed, recent studies by numerous groups have shown that the mitochondrial apoptotic pathway and death receptor pathway^15,21,38^ are also involved in GSDME activation and pyroptosis induction. In our case, we observed the cleavage of both GSDME and PARP depended on the activation of caspase-3, indicating CAP induced-apoptosis and pyroptosis were triggered by the same upstream pathway. In addition, our data showed CAP treatment activated caspase-9 but not caspase 8 (Fig. 4e), then activated caspase-3, and in turn cleaved GSDME and PARP. These results indicated that CAP-induced apoptosis and pyroptosis were mediated by the mitochondrial pathways. This conclusion was further supported by the activation of JNK and the release of cytochrome c from mitochondria (Fig. 5h). This finding was in agreement with our previous report that CAP-induced ROS activated the JNK/cytochrome c/caspase-9 pathway to trigger apoptosis^42^. Thus, mitochondria may act as a mediator between the CAP-induced ROS and pyroptosis/apoptosis.

The function of GSDME as the switch of apoptosis to pyroptosis has been recently studied. Overexpression of GSDME in HeLa cells result in switching apoptosis to pyroptosis after doxorubicin or 5-fluoruracil (5-FU) treatment^26^. Consistently, we also found that apoptosis was replaced by pyroptosis after CAP treatment in H1299 cells with exogenous GSDME overexpression, further confirming the key role in the apoptosis-to-pyroptosis switch. Recent studies further demonstrated that the expression level of GSDME was closely related to chemotherapy resistance and cell death^31,43–46^. Loss of GSDME promoted the resistance to cisplatin in lung cancers^38^. At the same time, a decrease in GSDME mRNA expression level contributed to increased etoposide resistance in melanoma cells^31^. On the contrary, reversal of GSDME silencing could sensitize tumor cells to doxorubicin and actinomycin-D^29^. Importantly, overexpression of GSDME has been observed in a subset of ESCC patients and correlated with a better prognosis, validating the clinical significance of GSDME^22^. Similar to previously reported chemical stimulation, our study showed the basal level of GSDME was also tightly associated with the sensitivity of tumor cells to CAP, which is a physical treatment. A more pronounced decline in viability after CAP treatment was observed in cells with high expressed GSDME compared with GSDME low expressed cells. In addition, we further confirmed the key role of GSDME in sensitivity of cells to CAP by knocking down or overexpressing GSDME (Fig. 3). Of note, further analysis of cell death following CAP exposure showed GSDME knockdown mainly led to reduction of pyroptosis cells, may be contributing to the sensitivity to CAP treatment. One possible reason may be the GSDME-mediated switch of apoptosis to pyroptosis, a more rapid cell death compared to apoptosis^25,26^. However, further studies will be needed to explore the specific molecular mechanisms. Together, our results indicated that the basal expression level of GSDME in tumor cells was closely related to CAP sensitivity and GSDME might be a potential biomarker of prognosis in the forthcoming cancer CAP treatment. These findings could provide a strategy to optimize the effect of CAP treatment.

**Fig. 6:**
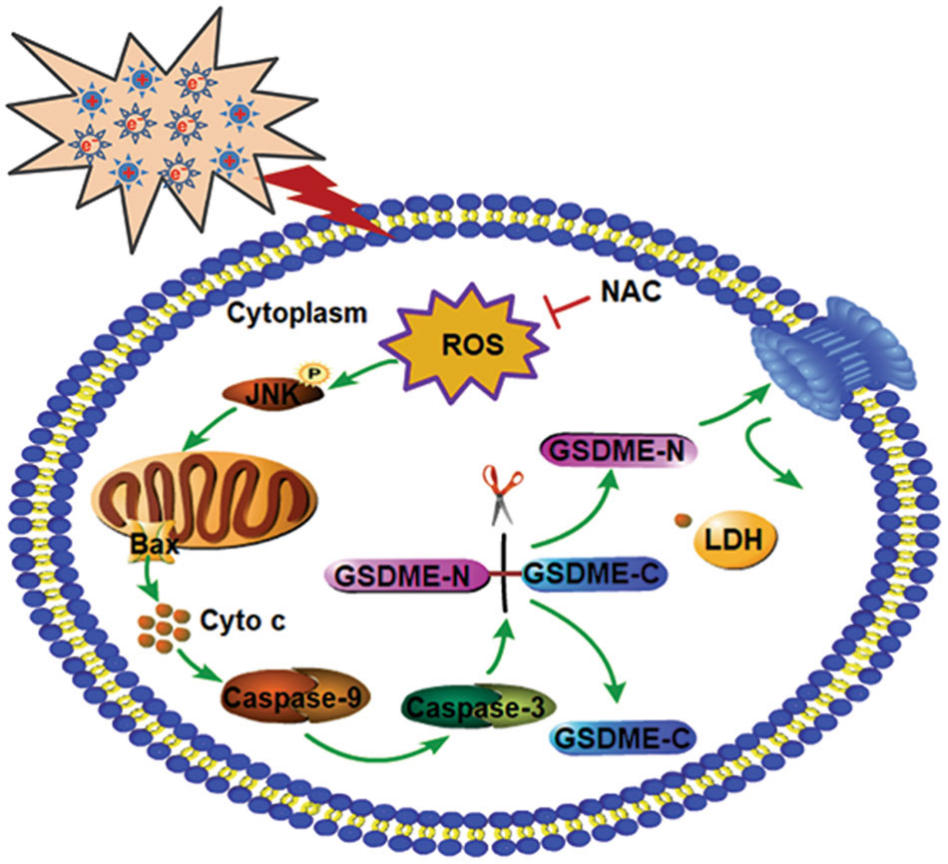
A pyroptosis pathway scheme of ROS/JNK/Cytochrome c/Caspase9/Caspase3/GSDME upon CAP treatment. In GSDME high-expressed cancer cells, CAP-induced ROS triggers JNK activation, translocation of Bax to mitochondria, release of mitochondria cytochrome c, and then activates caspase-9, which in turn activates caspase-3. This activated caspase-3 further cleaves GSDME, and eventually causes a typical pyroptosis morphology (cell swelling and membrane rupture) and LDH release. This process can be repressed by NAC.

In summary, GSDME-dependent pyroptosis was revealed in CAP treated tumor cells, and mechanism study illustrated that the apoptotic pathway, ROS/Caspase-9/Caspase-3/GSDME, was activated in GSDME high-expressed tumor cells to initiate pyroptosis (Fig. 6). Further studies should be performed to explore more mechanisms. It is anticipated that our study is helpful to cancer CAP therapy in the future.

## Supplementary information

Supplement Figure legends

Supplement Figure 1

Supplement Figure 2
