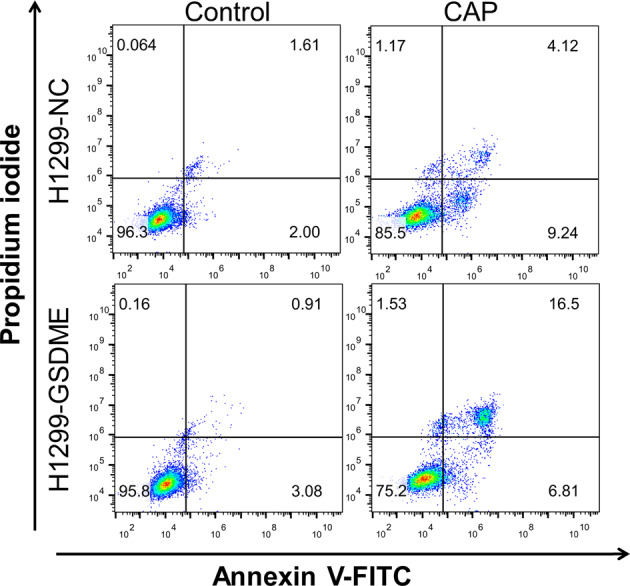# Correction: Cold atmospheric plasma induces GSDME-dependent pyroptotic signaling pathway via ROS generation in tumor cells

**DOI:** 10.1038/s41419-022-05143-7

**Published:** 2022-08-08

**Authors:** Xiaorui Yang, Guodong Chen, Kwan Ngok Yu, Miaomiao Yang, Shengjie Peng, Jie Ma, Feng Qin, Wei Cao, Shujun Cui, Lili Nie, Wei Han

**Affiliations:** 1 Anhui Province Key Laboratory of Medical Physics and Technology/Center of Medical Physics and Technology, Hefei Institutes of Physical Sciences, Chinese Academy of Sciences, Hefei, Anhui China; 2 University of Science and Technology of China, Hefei, Anhui China; 3 Department of Physics, City University of Hong Kong, Tat Chee Avenue, Kowloon Tong, Hong Kong; 4 State Key Laboratory in Marine Pollution, City University of Hong Kong, Tat Chee Avenue, Kowloon Tong, Hong Kong; 5 Clinical pathology center, The Fourth Affiliated Hospital of Anhui Medical University, Hefei, Anhui China; 6 Collaborative Innovation Center of Radiation Medicine of Jiangsu Higher Education Institutions and School for Radiological and Interdisciplinary Sciences (RAD-X), Soochow University, Suzhou, Jiangsu China

**Keywords:** Cancer therapy, Cell death

Correction to: *Cell Death and Disease* 10.1038/s41419-020-2459-3, published online 27 April 2020

The original version of this article unfortunately contained an error. During the result collation, the incorrect graphs were used in Figure 3H since a large number of graphs were processed at the same time. The authors apologize for the error. The corrected figure can be found below.