# Supplementary material for: Cold atmospheric plasma induces GSDME-dependent pyroptotic signaling pathway via ROS generation in tumor cells
Source: Cell Death Dis. 2020 Apr 27;11(4):295. doi: 10.1038/s41419-020-2459-3 (PMC7186223; doi:10.1038/s41419-020-2459-3)
Supplement: Supplementary file 1 — Supplement Figure legends [file 41419_2020_2459_MOESM1_ESM.docx]

**Supplementary Figure legends**

**Supplementary Fig. 1.** Apoptosis significantly induced in H1299 and MKN28 cells after CAP exposure （60 s）

**Supplementary Fig. 2.** Cell viability in PC9 and SGC-7901 cells pre-treated with pan-caspase inhibitor zVAD (30 µM) for 2 h following CAP exposures (40 s).
