# Supplementary figures and images for: Cold atmospheric plasma induces GSDME-dependent pyroptotic signaling pathway via ROS generation in tumor cells
Source: Cell Death Dis. 2020 Apr 27;11(4):295. doi: 10.1038/s41419-020-2459-3 (PMC7186223; doi:10.1038/s41419-020-2459-3)

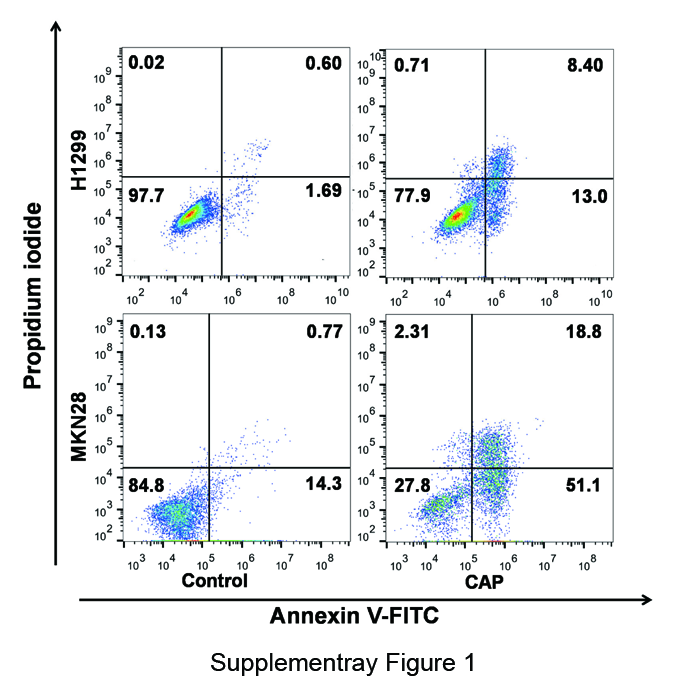

Supplement: Supplementary file 2 — Supplement Figure 1 [file 41419_2020_2459_MOESM2_ESM.tif]

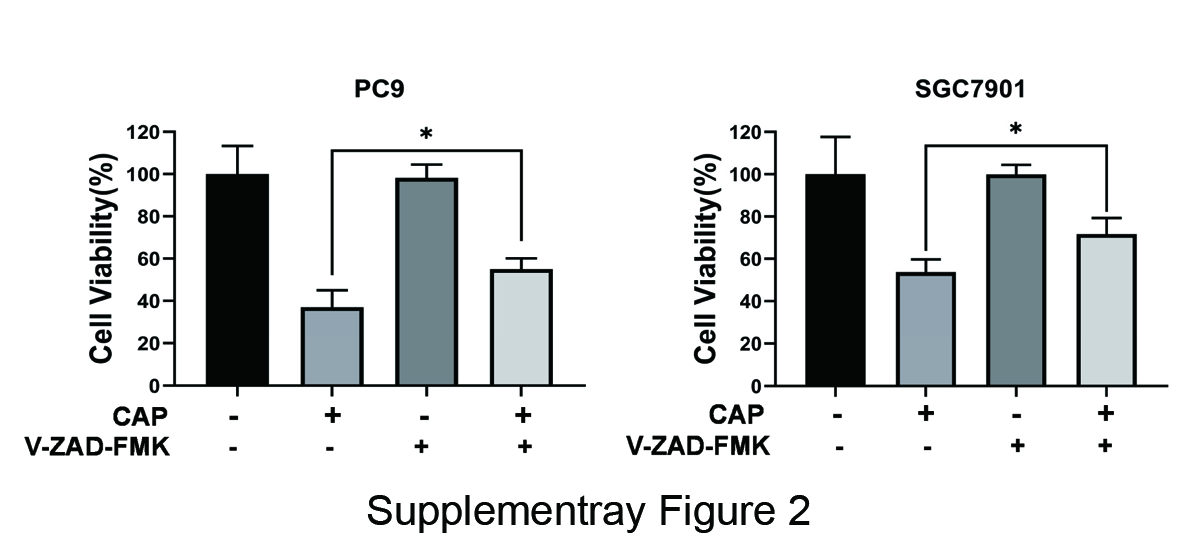

Supplement: Supplementary file 3 — Supplement Figure 2 [file 41419_2020_2459_MOESM3_ESM.tif]
